# Hyperglycemic Hemichorea: A Case Report

**DOI:** 10.7759/cureus.39240

**Published:** 2023-05-19

**Authors:** Joana Lopes, Eulália Antunes, Bárbara Oliveira, Vânia Gomes, Sofia Caridade

**Affiliations:** 1 Internal Medicine, Hospital de Braga, Braga, PRT

**Keywords:** decompensated diabetes, involuntary movements, diabetes mellitus in elderly, hemiballismus, hemichorea, hyperglycemic hemichorea

## Abstract

Chorea is a hyperkinetic movement disorder characterized by a continuous flow of rapid, random, and involuntary bodily contractions, usually affecting the distal limbs. When these movements are more proximal or assume a larger amplitude with a flinging or kicking character, they’re referred to as ballism. These disorders can be associated with several causes, ranging from genetic and neurovascular to toxic, autoimmune, and metabolic. Non-ketotic hyperglycemic hemichorea-hemiballismus is a rare consequence of decompensated diabetes mellitus with a poorly understood pathogenesis but with characteristic MRI T1 and T2 hyperintense abnormalities in the contralateral basal ganglia.

We present the case of a 74-year-old woman with a history of poorly controlled type 2 diabetes mellitus, dyslipidemia, and arterial hypertension who was admitted to the emergency room due to a two-day history of rapid non-stereotypical involuntary movements of the left side of her body. A neurological exam showed large amplitude and repetitive left-side body movements. Glycemia was 541 mg/dL with no ketosis. Her glycosylated hemoglobin was 14%. A brain CT excluded acute abnormalities. Brain MRI showed a discrete T1 hyperintense signal involving the right corpus striatum, compatible with non-ketotic hyperglycemic hemichorea-hemiballism syndrome. After metabolic optimization with insulin and haloperidol, the movements resolved. Early recognition and metabolic control are essential to the resolution of choreiform movements. Our aim is to raise awareness for hyperglycemic hemichorea-hemiballismus, in which decompensated diabetes is the early sign of diagnosis.

## Introduction

Chorea is a syndrome characterized by the continuous flow of rapid and involuntary body muscle contractions. When they are more severe and violent, they are referred to as ballism. As opposed to the usual distal and low-amplitude contractions in chorea, ballism movements are more ample and proximal [1, 2]. Non-genetic chorea can be divided into different etiological types: vascular, autoimmune, drug-induced, metabolic, and infectious [1, 2]. Severe non-ketotic hyperglycemia is a rare cause of metabolic hemichorea or hemiballismus, most commonly found in poorly controlled elderly diabetic patients. The most common imaging pattern is evaluated by cerebral magnetic resonance imaging (MRI) and consists of a T1-hyperintense signal in the contralateral basal ganglia that usually improves within days after glycemic control [3].

## Case presentation

A 74-year-old woman with a previous medical history of arterial hypertension, dyslipidemia, obesity, and type 2 diabetes mellitus with poor metabolic control was referred to the emergency department. Alongside these comorbidities, the patient had a one-year history of memory impairment that affected her daily activities and adherence to oral medication. The patient complained of involuntary movements of the left hemicorpus that had begun two days prior and predominantly affected her left arm. Besides the involuntary and repetitive left-side movements (Video 1), the remaining neurological and general exams were unremarkable.

**Video 1:** Choreiform movements of the left hemicorpus.

No other symptoms were reported, namely altered mental status, dyspnea, chest or abdominal pain, or urinary or gastrointestinal symptoms. Her vital signs were normal. The patient had capillary blood glucose of 541 mg/dL, capillary ketones of 0.3 mmol/L, and glycosylated hemoglobin (HbA1c) of 14%. Arterial blood gas showed no acid-base disorders. Her other laboratory studies are disclosed in Table 1.

**Table 1:** The patient's laboratory results HIV: human immunodeficiency virus; HBV: hepatitis B virus; HCV: hepatitis C virus; EBV: Epstein-Barr virus; CMV: cytomegalovirus; Ig: immunoglobulin; VDRL: venereal disease research laboratory; AST: aspartate transaminase; ALT: alanine transaminase; T4: thyroxine

Parameter	Value	Reference values
Hemoglobin (g/dl)	14.1	11.9–15.6
Leukocytes (/μl)	5,800	4,000–11,000
Platelets (/μl)	145,000	150,000–450,000
Glycosilated hemoglobin (%)	14	3.4-5.8
Urea (mg/dl)	39	19–49
Creatinine (mg/dl)	0.9	0.6–1.2
Sodium (mmol/l)	132	135–145
Potassium (mmol/l)	4.2	3.5–5.1
Total bilirubin (mg/dl)	0.58	0.2-1.1
AST (U/l)	26	12-40
ALT (U/l)	16	7-40
Gamma-glutamyl transferase (U/l)	19	< 38
Alkaline phosphatase (U/l)	63	46-116
Total protein (g/dl)	4.3	5.7–8.2
Albumin (g/dl)	4.0	3.4–5.0
Corrected total calcium (mg/dl)	9.4	8.3-10.6
Magnesium (mg/dl)	17	16-26
Phosphorus (mg/dl)	3.1	2.4-5.1
C-reactive protein (mg/l)	4.40	<5.0
Ceruloplasmin (mg/dl)	31	20-60
Free T4 (ng/dl)	1.07	0.89-1.76
Thyroid-stimulating hormone (uUi/ml)	5.23	0.55-4.78
Folic acid (ng/ml)	4.9	>5.38
Vitamin B12 (pg/ml)	444	211-911
Ethanol (g/L)	0.04	
IgA (mg/dl)	203	40-350
IgG (mg/dl)	772	650-1600
IgM (mg/dl)	93.4	50-300
Serum protein electrophoresis	No monoclonal spikes	
Anti-nuclear antibodies	1/80 (negative)	
Borrelia	Negative	
HIV/HBV/HCV	Negative	
VDRL (syphilis)	Negative	
EBV/CMV	Negative	
Antineuronal antibodies	Negative	

**Figure 1:**
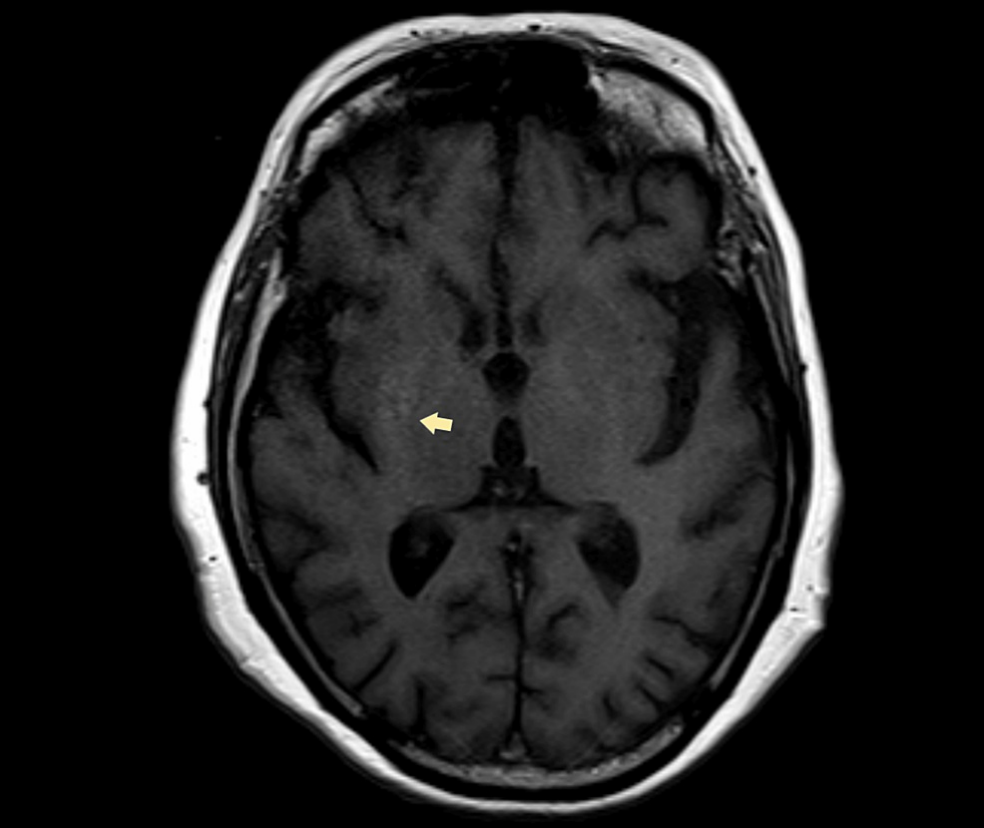
Discrete hypersignal involving the right corpus striatum (arrow).

Infectious serologic tests for borreliosis, human immunodeficiency virus (HIV) I and II, hepatitis B and C, cytomegalovirus (CMV), and Epstein-Barr virus (EBV) were normal. The autoimmunity panel, including antinuclear antibodies (ANA) and antineuronal antibodies, was negative. Seric protein electrophoresis, immunoglobulin A, G, and M, and ceruloplasmin were also normal. Subclinical hypothyroidism and a minor folic acid deficiency were detected, and the latter was promptly treated. A cranial computerized tomography (CT) scan showed no signs of acute ischemia or hemorrhage. Magnetic resonance imaging (MRI) showed a discrete hypersignal in T1 involving the right corpus striatum with no restricted diffusion (Figure 1). The hyperkinetic movements showed great improvement with insulin-based metabolic control and haloperidol.

## Discussion

Chorea is a rare hyperkinetic disorder characterized by irregular, rapid, and unilateral movements of the limbs, usually affecting the distal portion [3]. When focalized in the hemicorpus, the term hemichorea is used. Hemiballismus, on the other hand, refers to movements that are more proximal and of larger amplitude. Although less frequent involvement of the face and trunk can be seen [4]. There are various causes of chorea that can be categorized as hereditary (Huntington’s disease, spinocerebellar ataxias, Wilson’s disease, and X-linked syndromes), acquired (vascular, metabolic, and autoimmune), drug-induced, infectious, and structural [1]. Metabolic causes include hyperglycemia, usually in elderly women with longstanding and poorly controlled type 2 diabetes mellitus, which was the case of our patient [1]. The brain structures usually implicated in this disorder include the thalamus, subthalamic nucleus, caudate nucleus, putamen, and their interconnecting pathways [4]. Several disease mechanisms have been proposed, namely how the hyperosmotic state seen in decompensated diabetes promotes the breakdown of the blood-brain barrier, allowing the increased passage of white blood cells through the capillaries and subsequent lesions [4]. Ischemia of gamma-aminobutyric acid (GABA) pathways due to perfusion abnormalities secondary to hyperglycemia was also proposed, seeing as GABA has an important role in the inhibition of excitatory stimulation [4]. Typical findings in neuroimaging by brain magnetic resonance (MRI) include high-intensity T1-weighted and low-intensity T2-weighted lesions of the contralateral basal ganglia. Positron emission tomography (PET) with 2-deoxy-2-[fluorine-18] fluoro-D-glucose (18F-FDG) is also an option, showing a reduction in cerebral glucose metabolism in the basal ganglia on the affected side [3-5]. Our patient had a long history of poor metabolic control, with an HbA1c of 14% and non-ketonic hyperglycemia of 541 mg/dL. Although the usual presentation entails unilateral involvement, less than 10% of these cases have bilateral basal ganglia affliction [5]. Autoimmune, infectious, and toxic causes were ruled out, and there was no family history of chorea-like symptoms. Neuroimaging showed the characteristic findings in T1-weighted sequences involving the right corpus striatum. The absence of restricted diffusion excludes vascular causes. Insulin was initiated with good glycemic control. Haloperidol was also started because dopamine receptor antagonists can aid in chorea-like movement control in cases of severe motor symptoms [5]. Haloperidol was slowly reduced as glycemic control was achieved. Active improvement was seen after metabolic control; nonetheless, in some cases, symptoms can take up to weeks or even months to disappear [4].

## Conclusions

Severe non-ketotic hyperglycemia is a rare neurologic manifestation of poorly controlled diabetes mellitus. This entity is part of a long list of complications derived from diabetes mellitus, such as retinopathy, peripheral artery disease, neuropathy, and kidney disease, and it can sometimes be overlooked. Although poorly understood in its pathophysiology and dependent on strict metabolic control, the prognosis is overall good.
